# Immunocontraceptive Efficacy of Native Porcine Zona Pellucida (pZP) Treatment of Nevada’s Virginia Range Free-Roaming Horse Population

**DOI:** 10.3390/vaccines12010096

**Published:** 2024-01-18

**Authors:** Martin L. Schulman, Nicole K. Hayes, Tracy A. Wilson, John D. Grewar

**Affiliations:** 1 Veterinary Population Management Laboratory, Faculty of Veterinary Science, University of Pretoria, Pretoria 0110, South Africa; jdgrewar@gmail.com; 2 American Wild Horse Campaign, Davis, CA 95617, USA; nhayes@americanwildhorse.org (N.K.H.); twilson@americanwildhorse.org (T.A.W.); 3 JDATA, Sandbaai 7200, South Africa

**Keywords:** porcine zona pellucida, pZP, vaccine, free-roaming horses, Virginia Range

## Abstract

In North America, range constraints due to burgeoning development increasingly encroach on wild horse habitat and necessitate effective but humane reproductive management. The largest free-roaming wild horse fertility control program by population (>3500) and territory size (≈300,000 acres) is located within Nevada’s Virginia Range. Data from a field study investigated porcine zona pellucida (pZP) immunocontraception via remote dart delivery to mares in this population. Analyses aimed to measure efficacy by treatment effects on annual birth rates and population demographics and to evaluate treatment frequency and season against these variables. Analyses included mares’ monthly data (January 2019–December 2022; 48 months), characterized by cumulative vaccination numbers subset into four classifications considering the vaccine as having no loss of efficacy or a loss within a 6-, 12-, and 18-month period post vaccination; from foaling data, the likelihood of being in foal and of conceiving in that month; and from age, as mature or immature (<1 years-old). A downward foaling rate and trend in the numbers of mature mares, descriptively presented at monthly intervals, showed markedly declining annual seasonal breeding peaks, with no observed change in foaling season or duration. Within four years, population coverage surpassed 70% and was associated with a 58% reduction in foaling, with only a 10% conception rate. Vaccinated mares increased proportionally: assuming a 12-month decay rate, the system reached stability at an average ≈1.0 vaccination/mare/year, providing a robust recommendation for treatment frequency contributing to best management practices.

## 1. Introduction

In the United States of America (USA), the free-roaming wild horse or mustang has iconic status with both historical and cultural associations [1,2]. Free-roaming wild horses and burros are amongst a few species, including bald and golden eagles and the bison, to have species-specific dedicated federal legislation regarding their protection and, or historical significance in the USA [3,4,5]). Many of these populations of free-roaming horses in the USA have, however, arguably received insufficient managemental interventions to address important considerations. These considerations include habitat restriction and loss, overabundant cattle grazing on public lands, climate change, overexploitation of natural predators and drought [6,7,8,9]. This widely perceived inadequacy in responsive management has increasingly prompted controversy and public debate, particularly regarding various proposed population management strategies [7,10,11,12,13]. Increasingly, humane and effective management of free-roaming horse populations has become a concern and debated topic universally, including populations in Australasia, North and South America, Europe, and Africa [14,15,16,17,18,19].

Immunocontraception using various vaccine formulations that induce infertility or subfertility via stimulation of the immune system to produce antibodies to a component critical to the reproductive process is an established method for minimally invasive and effective population management [20]. Immunocontraception targets various antigens, including the oocyte’s zona pellucida proteins (ZP), to prevent sperm penetration for fertilization. Porcine ZP proteins (pZP) are derived from pig ovaries and have been used to formulate a native pZP vaccine, which has been successfully applied for contraceptive use in many species [21,22].

The contraceptive efficacy of pZP as an immunocontraceptive agent is well established [23,24,25,26]. The efficacy of pZP in equids was first demonstrated by Liu and others in 1989 [27]. The first in-the-field application of a native pZP vaccine to manage a wild horse population commenced in 1988 at Assateague Island National Seashore (ASIS) in Maryland, USA [28]. The vaccine, Zona-Stat H, produced by the Science and Conservation Center in Billings, Montana, is registered by the US Environmental Protection Agency (EPA). The vaccine comprises pZP proteins formulated together with one of either Freund’s complete adjuvant (FCA) or Freund’s incomplete adjuvant (FIA) to stimulate an immunogenic response in target animals. Following immunization, fertilization is prevented. This fulfills one of the critical postulates required of an effective contraceptive agent without interference with the target animal’s reproductive cyclicity and behaviors [24,28]. This downstream effect was of particular importance in species, including *Equidae ferus,* whose gonadal steroid-driven behaviors and herd integrity are dependent on ongoing reproductive cyclicity [22]. Although decreased female band fidelity may occur with immunocontraception, it was recently reported that male fecal cortisol metabolite concentrations did not vary with increased female turnover [29,30]. The successful application of pZP at ASIS continues to maintain this population at target levels more than three decades since inception and has supported the safety and efficacy profile of this treatment. The application of this vaccine was extended to the western USA in the 1990s as a treatment for wild horses and burros. In free-roaming horses in Nevada, successful immunocontraception was reported with a significant effect on suppression of foaling rates using pZP vaccination with various formulations and administration frequencies [25,26].

Despite pZP vaccine’s relatively widespread and successful application, various important aspects of this method remain incompletely defined. These include considerations in selecting pZP as a population management tool, particularly for its application in the larger wild horse populations inhabiting extensive ranges in the western USA [31]. Reportedly in these habitats, reliable access to the target population’s mares poses significant limitations on a program’s contraceptive success [32]. These limitations pose an important challenge to enabling the continual and extended application essential to successful results at the population level [33]. The effective administration of the Zona-Stat H vaccine requires an initial course of a primer and booster vaccination, maintained subsequently with annual booster treatments. Although clearly successful in its application in environments and populations such as ASIS, the protocol poses significant economical and logistical challenges in the more extensive and varied environments characterizing the western USA. As early as 1990, Kirkpatrick and Turner advised a single-dose formulation as an essential prerequisite to a program in extensive habitats [28]. That application for pZP formulations was additionally limited by the need to capture and confine horses to allow hand delivery [25,33]. A previous study in the Virginia Range observed a smaller area with hand treatment of a population of captured horses with two immunocontraceptive vaccines, one a long-acting pZP formulation [25]. Variable individual efficacy was reported, suggested to be both associated with body condition and likely to be enhanced in captive as compared with free-ranging subjects [34]. It was additionally concluded that vaccination as a strategy was limited by practicalities inherent in annual treatments and the need to capture mares.

Remarkably, despite the generally accepted contraceptive efficacy of pZP vaccines via remote darting in wild horses, there is a paucity of reports investigating the efficacy and evaluating the feasibility of immunocontraception programs applied in larger populations of wild horses. Most of the reported research has focused on smaller horse populations in limited habitat areas [23,26,33,35,36].

The American Wild Horse Campaign (AWHC), a non-profit organization, entered into a cooperative agreement with the Nevada Department of Agriculture (NDA) to treat free-roaming horses residing within the area referred to as the Virginia Range located outside Reno, Nevada, USA. This habitat is increasingly impacted by the burgeoning of commercial and residential development, creating an urgent need for humane population control. The program’s aim was to treat breeding-age mares using a native pZP vaccine via ground-delivered darting and commenced in March 2015. In October 2017, however, the State of Nevada halted the program. This permission was subsequently reinstated, and the current program commenced on 9 April 2019. With approximately 3500 horses inhabiting the 300,000-acre habitat, this is almost certainly the largest fertility control program for wild horses, both in terms of territory size and population numbers.

A field study observing a large, free-roaming population inhabiting an extensive range over an extended period would provide a unique opportunity to compare previously reported observations regarding efficacy and, importantly, to investigate the feasibility of dart-delivered pZP immunocontraception in wild horses. The objective of this initial study, reported here, was to analyze data for the treatment protocol over four years (2019–2022) to provide a model to measure efficacy by observing the vaccine’s effects on annual birth rates and population demographics in a large population inhabiting an extensive range. This study’s findings would support the feasibility of this method as a practical, humane, and minimally invasive method for the population management of wild horse herds in this environment in the western USA.

## 2. Materials and Methods

### 2.1. Virginia Range

The Virginia Range is situated in western Nevada and constitutes ≈300,000 acres located to the east of the city of Reno. This mountainous range is part of the drainage divide between the Truckee River in the north and the Carson River in the south and is bounded by highways and interstates on all sides. The elevation across this range varies between 1370–2370 m at Mount Davidson. This habitat is a semi-arid high-elevation desert, where the flora consists of sagebrush (*Artemisia tridentata*), rabbitbrush (*Chrysothamnus nauseosus*), and a variety of grasses. Trees, including Jeffery pine (*Pinus jefferyi*), some pinyon pine (*Pinus monophylla*) and junipers (*Juniperus utahensis*), are found at higher elevations. Other ungulate species occupy the range, including mule deer (*Odocoileus hemionus*), pronghorn antelope (*Antilocapra americana*), and desert big horn sheep (*Ovis canadensis nelson*).

### 2.2. Free-Roaming Horse Population

**Figure 1:**
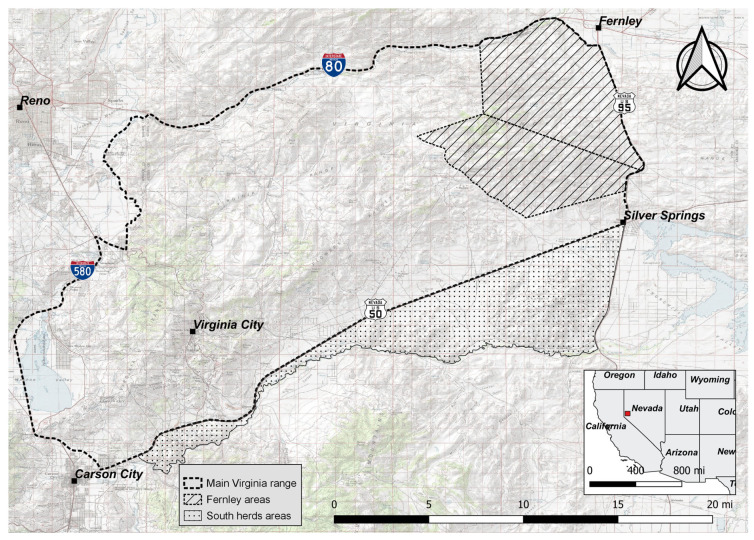
Map of Virginia Range, Nevada, USA, indicating the main Virginia Range (no fill), Fernley areas (line fill), and south herd areas (dot fill).

The free-roaming horse population that inhabits the Virginia Range resides primarily on private land, with some public land interspersed, and is managed through the NDA. As this area is predominantly private land, development is continuously removing available rangeland. The currently ongoing Virginia Range fertility control program commenced on 9 April 2019. The study investigated data sourced from the current program for the 48 months observed from 1 January 2019 through 31 December 2022. Although the Virginia Range is bound in the south by the Carson River, darting permission for the program is valid only to Highway 50. Highway 50 is unfenced for large portions in addition to wildlife underpasses, and thus horses can cross back and forth. This population of horses south of Highway 50 remains predominantly untreated due to these restrictions (south herd areas, Figure 1). Additionally, the far eastern edge of the range (Fernley areas, Figure 1) did not begin documenting and darting effectively until 2022 once the program had expanded to include these areas.

### 2.3. Horse Identification and the Database

The AWHC has maintained a database for the Virginia Range horse population since 2015. This was continuously updated based on field observations reported by volunteers. Horses were individually identified using herd treatment area, band affiliation, gender, and horse color/markings as recorded in the database. As of 31 December 2022, there were individual records for 5453 horses (characterized as living (n = 3418), removed (n = 54), and deceased (n = 1981). Deceased horse count includes historical records of horses documented prior to program’s inception in 2015 that were added for genealogical tracking. Within the database, individual horse records contained identifying color photographs (left side, right side, face, leg markings, scars, etc.), coat color, gender, distinctive markings, date of birth when known, location, history of band changes, births, and the dam and sire, when known, in addition to any injuries or other condition notes. Foals were assigned to the dam based either on affiliative behaviors or when the birth was witnessed. All foals were assigned a date of birth based on appearance of individual foals when first observed and individual mare’s final observation before parturition.

### 2.4. In-the Field Darting Teams and Coordination with Delivery and Data Recording

Horses were monitored and darted by a team of experienced volunteers comprising 18 darters and 21 documenters. The vaccine was delivered remotely by darters that had undergone training with subsequent certification by the Science and Conservation Center (SCC) in Billings, MT. Documenting and darting activities occurred year-round to encompass all seasons. All observations, records, and treatments were updated in the database on the same day as observation or treatment. Within each mare’s record, the fertility control applications were recorded and included the type of action (primer or booster), date, side of the horse darted, distance of the darting shot, amount of CO_2_ used, number and type of dart used, type of delivery method, observed treatment reactions, such as abscesses or granulomas, and the darter’s identity. Each day prior to darting, in-the-field teams accessed a report that lists individual mares requiring vaccination within a selected area. The team would then explore the area and opportunistically dart and concurrently record any mare identified as requiring treatment.

### 2.5. Vaccine Formulation, Storage and Delivery Method

Horses were all treated with a native pZP vaccine (Zona-Stat H, Billings, MT, USA). The vaccine was packaged as 100μg doses constituted as a 0.5 mL volume in 1.0 mL vials. This native vaccine was shipped from the SCC in Billings on dry ice and subsequently stored frozen at −20 °C. The adjuvants for inclusion in the vaccine formulation were provided by the SCC, and these included modified Freund’s complete adjuvant (mFCA) and Freund’s incomplete adjuvant (FIA), which were both stored refrigerated at approximately 5 °C.

### 2.6. Vaccination Protocol

All mares older than eight months of age were allocated for treatment via loading darts with the vaccine for remote delivery. For mares never previously vaccinated, the initial (primer; P) treatment consisted of 0.5 mL pZP vaccine emulsified prior to administration with 0.5 mL mFCA. The second (booster; B) treatment was administered ≥2 weeks post-P or repeat primer (RP) and consisted of 0.5 mL pZP vaccine similarly emulsified with 0.5 mL FIA. The initial course is followed within 8–12 months and repeated thereafter with a B treatment of 0.5 mL pZP vaccine emulsified with 0.5 mL FIA. The treatment course was restarted (with administration of a repeat primer (RP) and B) if a mare (a) was primed and never boosted after ≥12 months; (b) had a foal or appeared obviously pregnant with no recorded treatment omissions; and (c) had an interval ≥14 months between B treatments, according to the mare’s darting records.

Once a mare had been positively identified and was confirmed to require vaccination, the darter would thaw one vial of pZP to be emulsified with the appropriate adjuvant (for either one of P, RP, or B). The emulsion was then loaded into darts for delivery via a 1.0 mL Pneu-Dart^®^ with 1.5” barbless needles discharged from either Dan Inject^®^ (delivery ≤ 45 m) or Pneu-Dart^®^ X-Caliber (delivery ≤ 70 m) rifles, respectively. Safety for both operators and horses was an important consideration. All darting teams were required to wear high-visibility safety vests when in the field. Mares were darted on properties with both owner and final approval by the NDA. Darting was prohibited within 500 feet of roadways, residences, or commercial buildings. No attempts to dart were made in the following conditions: high prevailing winds; ranges exceeding the rifle’s capability; when the horse was standing at an angle, risking the dart missing the hip/gluteal region and striking the thorax; when a foal was adjacent or nursing a mare; or when there was a risk of the dart missing and impacting another animal. The ideal position was the darter standing perpendicular to the targeted mare at a 90° angle to the hip or gluteal region. Once a shot was taken, the dart would drop from the mare. This was then gathered, thus enabling confirmation that the dose was administered by checking that the dart plunger had engaged to discharge the vaccine prior to disposal in an appropriate sharp’s container. If the dart did not immediately drop, mares were followed until the dart detached. Any visible splash on the coat or visible blowback from the dart was noted. If the dose was not confirmed to have been delivered, the attempt was labeled as a misfire, and the mare would subsequently be re-treated. An inventory control form for each darting event was included in the database. Any visible effect from darting is recorded in the database. These visible impacts include discharging abscesses and granulomas empirically characterized as discrete, raised nodular skin masses [31]. The date of first observation of any such lesion was recorded, and thereafter the date when this lesion was observed as having resolved was additionally recorded within the database and tracked for each mare.

### 2.7. Data Analyses

For the analysis, each female horse was assigned monthly data points based on their individual period of activity within the study window. These monthly data points served as temporal markers for each variable evaluated that included pre-2019 vaccination status, cumulative vaccinations (considering permanent, 6-, 12-, and 18-month vaccine efficacy); cumulative vaccination during the study period; pregnancy status; conception in month; foaled in month; maturity status; associated band; associated herd area; granuloma observed; and abscess observed. Each of these variables was recorded or computed for every monthly data point.

Horses located for any continuous 12-month period, within their enrollment, but outside the geographical limits prescribed by the NDA for darting permission (horses south of Highway 50, South herd areas, Figure 1) were removed from the analysis. A 12-month period was used for exclusion as this interval coincided with the end of the treatment window for boosting, and thus the mare’s treatment was considered to have expired. This exclusion criterion did not remove the horses that continually cross back and forth across the darting boundary, even though this may result in missed treatments. Additionally, horses that were resident in the far eastern edge of the range (Fernley areas, Figure 1) were excluded as they had only received one year of treatment during the study’s observation period, and thus efficacy data were unavailable. After this removal, individual records for 2817 female horses (characterized as living (n = 1706), removed (n = 22), and deceased (n = 1089) as of 31 December 2022 were included in analyses.

For this analysis, mares were separated between mature (>1 years-old) and immature (≤1 years-old), respectively, based on reproductive potential from >1 year of age previously reported in free-living horse populations in the western USA [37,38]. The program’s protocol had empirically elected to commence vaccination of mares ≥8 months of age to mitigate the risks associated with failure to reliably relocate a peripubertal mare prior to her becoming reproductively mature. Female horses for which a date of birth was not captured were considered as mature in their first month of evaluation in the study.

Four scenarios were created considering the uncertainty of the length of efficacy of the vaccination administered (considering permanent, 6-, 12-, and 18-month vaccine efficacy). The first scenario was permanent efficacy, in which case the number of vaccinations per mature mare accumulated incrementally from the start to the end of the study period for each vaccination administered. For each month of the study, the mean number of vaccinations per mature mare at that point was the mean cumulative vaccinations administered across the study dataset at that point in time. In decreasing order of efficacy, we then considered 18-, 12-, and 6-month efficacy. In the worst-case scenario (6-month efficacy), the cumulative count of vaccines administered per mature mare takes into consideration that the vaccine was only efficacious for 6 months. For example, a mare vaccinated for the first time in month 8 of the study would have a cumulative count of 1 vaccine for months 8–13 of the study and then revert to a cumulative count of 0 in month 14, which would remain until vaccinated again. For each month of the study, the calculation of the mean number of vaccinations per mature mare remained the same (as described in the permanent efficacy above) but also considers that the vaccine administered may not be efficacious throughout.

Total mares, total vaccinations, and total mares vaccinated was summed across each group of mares by month and year. Total observations of visible darting-associated lesions characterized as either granulomas (raised swelling) or abscesses (open and discharging pus) were summed by year, and the prevalence was determined by dividing occurrence by total number of vaccinations given in that year. Foal births were summed by month and year, and foal births were used as a proxy for previous year’s conception based on a gestation period of 354 days, with the assumption that the actual conception rates were likely higher due to undetected pregnancy attrition at any stage between fertilization and parturition.

All data were retained in a PostgreSQL database (www.postgresql.org/) (accessed on 25 July 2021) with postgis (https://postgis.net/) (accessed on 25 July 2021) enabled for management of spatial data. Herd area polygons were defined by AWHC employees. The roads and underlying basemap (USA Topo Maps) used in Figure 1 were sourced from National Geographic Society, i-cubed Copyright: © 2011—https://www.arcgis.com/home/item.html?id=99cd5fbd98934028802b4f797c4b1732 (accessed on 26 October 2023). Polygons depicting the states of the USA in the overview map in Figure 1 were obtained from Natural Earth (https://www.naturalearthdata.com/downloads/10m-cultural-vectors/10m-admin-1-states-provinces/) (accessed on 26 October 2023)) and are publicly available for use.

Basic count and summary statistics were performed using SQL. All detailed and graphical analysis was performed in R (version 4.1.0) using RStudio 2022.02.0+443 “Prairie Trillium” sourced from https://posit.co/products/open-source/rstudio/ (accessed on 23 June 2023) [39]. Exploratory data analysis was performed using the explore package [40]. Graphics were generated using ggplot2 [41]. Other R packages used for data management and manipulation included RPostgres, dplyr, and scales [42,43,44].

## 3. Results

**Figure 2:**
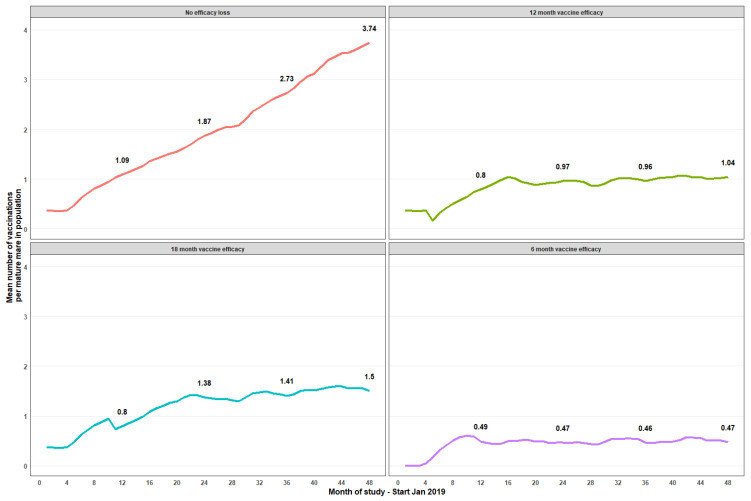
Temporal trend of mean pZP vaccinations per mature mare at monthly intervals across 48 months. Annotated labels show 12-month interval values. Panels indicate estimated variability of the vaccine efficacy period from no loss (top-left panel) through to an efficacy period of 6 months (bottom-right panel).

During the first year, treatments commenced in April 2019, and a total of 760/1528 (49.7%) mature mares were treated with a total of 1195 vaccine administrations. Subsequently, over 12 months of the second year, 1070/1794 (59.6%) mature mares were vaccinated with administration of a total of 1706 vaccines. By the end of this study’s observation period, in the course of the fourth year, 1257/1963 mature mares were treated with administration of 1821 vaccinations (Table 1). This represented 64% of all mature mares recorded in 2022; considering this fourth year alone, 1425 mares were treated with ≥1 dose, representing 72.5% of the population. Vaccination of immature mares ranged between 6 (2019) and 101 (2021) during the study (Table 1). Mean vaccinations per mature mare increased steadily across the program; by month 12, a mature mare averaged 1.09 vaccinations, reaching 2.04 (month 27), 3.06 (month 39), and 3.74 (month 48) at the end of the observation period with an assumption of no loss of vaccine efficacy (Figure 2). Assumptions of 6-, 12-, and 18-months efficacy showed averages of 0.5, 1, and 1.5 treatments, respectively, by month 48 of the program. Unsurprisingly, the greatest coverage was observed in the model assuming no efficacy loss, with a mean vaccination of 3.74 vaccinations/mare by month 48. With a modeled 12-month efficacy assumption, a mean ≈1 vaccination/mare commenced ≈month 16, being maintained ≈1 vaccination/mare until month 48 (Figure 2). With 6-month vaccine efficacy, mean vaccinations per mare resulted in a plateau ≈0.5 vaccination/mature mare at any point in time (Figure 2).

Observations of externally visible side effects of dart delivery included recording both granulomas and abscesses (Table 2). These lesions were infrequent, as reflected in a maximum annual occurrence in 2022 of 11 (0.59%) and 24 (1.28%) granulomas and abscesses, respectively, associated with 1821 vaccine administration events.

In 2019, 392 foal birth events were recorded, and this increased to 488 in 2020 (Table 3). In 2021, however, births were reduced by 45.3% to 267 and reduced further to 205 in 2022, representing a 58% reduction compared to 2020 (Table 3). Foal mortality included all deceased foals <1 years-old with death recorded within that year, even if the birth year was in the preceding year. Foal mortality increased from 2020 and peaked at 63% in 2022. This apparent increase in foal mortality across time was likely associated with increased range coverage and observation of foals before reaching 1 year of age. The sex ratio of foals was relatively even, with the exception of 2021 where it was slightly skewed towards females; however, a concurrently increased incidence of female foal mortality that year resulted in a relatively even foal crop (Table 3).

**Figure 3:**
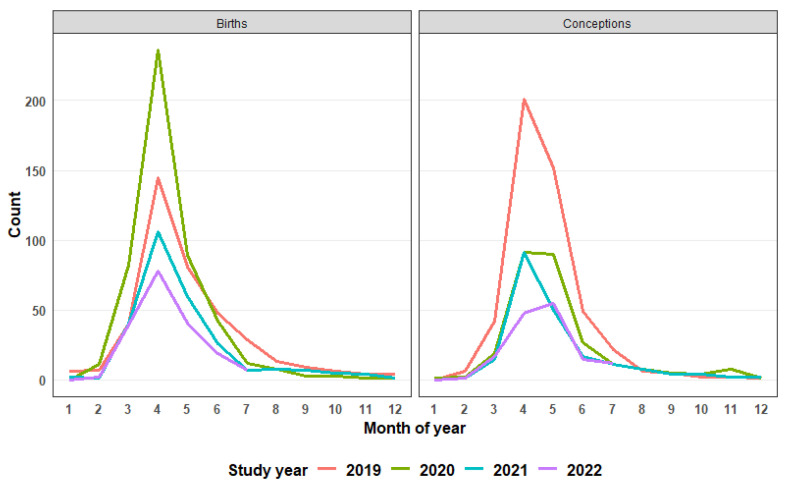
The number of foal births recorded by month and year (2019–2022) and with this number applied as proxy for conceptions in the previous year (assuming 354 days gestation) in mature mares.

**Figure 4:**
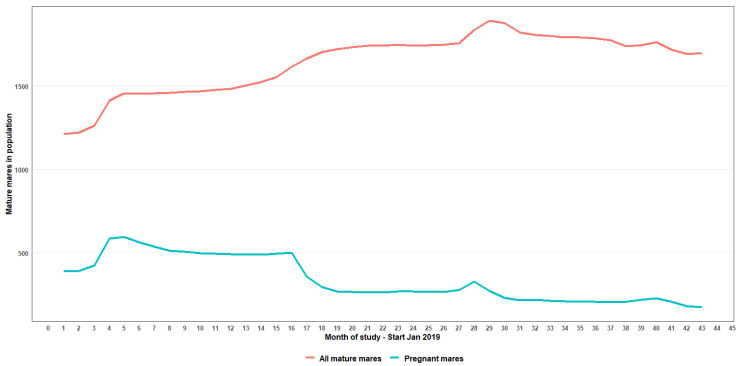
Graphical representation of the divergence of pregnant mares as a proportion of the mature mare population between 2019 and 2022.

The seasonal response of foal births (Figure 3) showed that subsequent to the increased rate recorded in 2020, there was a decline both in the peaks and, notably, a steady but marked temporal contraction year-on-year in this pattern. Additionally, no apparent widening of foaling season or shift in peak foaling season was observed (Figure 3). When foal births were used as a proxy for conceptions, in 2019, 488 mares conceived, comprising 31.9% of the mature mare population, and thereafter the proportion of mares conceiving year-on-year was more than halved to 14.9% (2020) and 10.3% in 2022 (Table 1, Table 3). The trend of total mares in the population along with their pregnancy status is shown in Figure 4. Even with improved observation resulting in an increase in the total mares reaching a peak in month 29 of the study, there was a decrease in the numbers of pregnant mares across the study period, with the trend still decreasing at the analysis end point of 48 months. The number of pregnant mares peaked in month 5 (n = 551) and sharply declined 67% by month 43 (n = 173). In addition, data showed a shallow but decreasing (10%) trend of total females in the population, commencing from month 29 of the study.

## 4. Discussion

By the end of the study’s observation period, ≈¾ of the mature mares had been vaccinated at least once, with individual mares averaging ≈4 treatments. During that final year, ≈⅔ of mature mares received treatment, with a foaling reduction of almost 60%. Through month 29, the mature mare population increased; however, this demographical change was associated with multiple factors including the natural maturation of mares and more notably, a steady increase in access to mares and expansion of the program’s coverage area. At inception, a darting program must first identify and document the subject population, which may serve to delay access to a larger number of mares. As horses are encountered and identified, with this information being added to a database, the additional information may create the artifact of an increased population rather than increase in documentation. In addition, with the passage of time, the data suggested both an enhanced recording efficacy and treatment administration by darting teams in the field. This arguably was a result of concomitant increased familiarity with the terrain and the target population, to be expected as a program expands.

Data demonstrated that increased population coverage was clearly associated with both declining reproductive and foaling rates. Treatment data showed a 35% increase yearly in vaccine administrations. Several reports have previously highlighted the critical importance of this population coverage as a predictor of success [33,37,45]. Our results supported the recently postulated “fertility control index”, as the product of the proportion of mares treated, and vaccine efficacy in these populations [33]. This index is predictive of both population growth and reproductive rates. Grams and others in 2022 further referenced that 60–90% of mares would need to be treated to achieve a stable population in the absence of removals [33]. The study’s current vaccination coverage falls within this range, and the yearly rate of increased administrations suggested that this strategic objective was a feasible goal. Furthermore, the current study’s observations suggested that sustained treatment delivery, stated as a critical criterion for success [32], was possible even in the large, extensively distributed population inhabiting challenging terrain. This contradicted the caveat that this method’s success was achievable only within small, discrete and easily accessed populations [46,47]. The Virginia Range itself was previously reported as impractical and cost-prohibitive for a multiple-year pZP vaccination program due to the large resident population and extensive area [48]. This program, however, achieved vaccination coverage within four years of ¾ of the mare population resident in a range of almost 300,000 acres. Importantly, this included mares that spent up to a year outside the approved darting boundary, which would have resulted in missed treatments. Furthermore, despite assertions that a single-dose formulation was an essential prerequisite in free-roaming horses, offering economic and logistic advantages [28,48,49], the current study’s results show its efficacy and feasibility despite its reliance on a multi-dose formulation.

Analysis of the efficiency of vaccination coverage when considering the observed reproductive and foaling outcomes showed that these demographic changes occurred even assuming a “worst-case” scenario (6-month vaccine efficacy) that results in a plateau of ≈0.5 vaccinations per mature mare at any point in time. Certainly, with a biologically plausible expectation of 12-month efficacy with its resulting average of 1 vaccination per mare, respectively, this would result in the observed demographic changes. Until recently, there were surprisingly few reported side effects of vaccination with immunocontraceptive agents [50]. This may reflect the limitations imposed on accurate clinical observation in targeted free-roaming wildlife populations, including injection site reactions and musculoskeletal effects, such as lameness subsequent to darting [50]. Similar to an earlier report [31], this study’s data showed a relatively low incidence and transient nature of observed lesions associated with pZP treatment. An anti-GnRH vaccine applied in horses was associated with a higher incidence lesion, albeit these were also transient in nature [51]. Various reports have implicated Freund’s adjuvants in vaccine formulations with both native and recombinant ZP antigens in association with injection site reactions in both horses [52] and donkeys [53,54], and there are currently reported investigations to find alternative adjuvants [53,55,56]. Globalization of native pZP vaccines is currently limited by factors including access to the antigen in many countries, animal-derived ZP antigen with associated contamination and biosecurity risks, and the economics of its production [36,54,57]. Recent reports on equids with administration of recombinant ZP antigens formulated with alternative adjuvants offers the potential for wider application.

There was a perceived increase in foaling rates in 2020, the second year of the program; however, due to incomplete documentation of the range, the first year (2019) was almost certainly an underestimation of the full foaling rate. Subsequently, foaling rates approximately halved in 2021 and by 2022 were further reduced by almost 60%. This was paired with a high foal mortality rate, peaking at 63% in 2022. At a program’s inception, especially at these geographic and population scales, it should be anticipated that time is required to develop and optimize observational efficiency. An equine gestation period of almost one year inherently implies that a variable proportion of the initially treated mare population were already pregnant at the time of initial vaccination [57]. This merits consideration for this study’s program as its regulatory approval was not granted until April 2019, which corresponded with peak foaling season. An advantage of pZP administration, however, is that it has no deleterious effect on a concurrent pregnancy in treated mares, and can thus safely be administered at any point during gestation [27,28]. Due to the prolonged equid gestation, an appreciable contraceptive result will necessarily show a lag until the second foaling season. Examples of zero-population growth targets include a study where 2 years were required even in the small, geographically contained population treated at Assateague Island, and a marked decrease was only apparent at 11 years. This slow rate was reportedly associated with advantages beneficial to animal welfare, including increased longevity, reduced mortality rates, and improved body condition of treated animals [58]. The current study’s observation period precludes comments regarding longevity and reversibility. Although not included in the database, subjectively the body condition of treated mares in this population was invariably classified as “good” via reference to the photographic and in-the-field observations. However, a population decrease might arguably be expected sooner when considering the Virginia Range population due to high mortality rates associated with documented predation [59].

When applying foaling rate as a proxy for conception rate and, arguably, inversely for contraceptive success, this showed that prior to 1 January 2019, 1/3 and by 31 December 2022, 1/10 of mares in the population, respectively, had conceived in the previous year. Importantly, no shift in conception seasonality was apparent, with no observed extension of the foaling season, as was previously suggested in other studies [60,61]. In association with the resultant impact on conception rates, the pregnant mare demographic in the Virginia Range population showed a distinct shift over the 48 observation months. The proportion of the total mare population that was pregnant had almost halved after two treatment years, and the number of pregnant mares declined 67% by the end of the study. Further reduction in contraceptive success is a reasonable expectation with the continuation of this program.

Interestingly, observations from this study population showed several similarities reported from the pZP immunocontraception program in elephant cows in South African reserves. Both programs exclusively utilized a single source of the native pZP vaccine from abattoir-derived pig ovaries with solubilized pZP proteins formulated with FMCA (primer) and FIA (booster) treatments [21,35,50,62]. Similarly, in both species characterized by prolonged gestation periods, initial results may appear disappointing. A high percentage of treatment coverage with sustained delivery were prerequisites for marked contraceptive efficacy. In the elephants, calving rates declined significantly following 3–5 years of treatment, and individual identification was highlighted for treatment success [50,62]. In addition to elephants, pZP immunocontraception has been shown to be successful for population management of bison, as well as an being effective contraceptive for >60 species [63,64,65]. The globalization of native pZP vaccines is currently limited by factors including access to the antigen in many countries, animal-derived ZP-antigen and associated antigen purity and biosecurity risks, and the economics of its production [36,54,62]. Recent reports in equids with administration of recombinant ZP antigens formulated with alternative adjuvants offer the potential for wider application.

## 5. Conclusions

There are, as frequently reported, undeniable challenges in applying immunocontraception to wild horse herds occupying the extensive, difficult-to-access terrain typical of the western USA. This study showed that sustained, multi-dose administration of a native pZP vaccine was able to significantly reduce growth rates of even a large and extensively distributed population inhabiting challenging terrain. Population coverage had reached 72.5% within four years, which resulted in an almost 60% reduction in foaling. This was possible despite the additional complication of mares missing vaccinations due to their movement outside the approved darting area. Even with the assumption of lowest efficacy, the proportion of mares vaccinated in this population had increased steadily. The more likely assumptions of some decline in efficacy, i.e., the intermediate scenarios, show that based on available data, the population coverage is currently somewhere between 40–80%, resulting in a conception rate of only 10%, with no shift or widening of foaling season. The most salient findings were as follows:

- The system reaches stability (even if adding mares to the population) assuming a 12-month decay rate ≈0–1.5 vaccinations, i.e., average ≈1.0 vaccinations/mare/year;
- Can recommend a target (assuming a 12-month decay rate) ≈1.0 vaccinations/mare/year;
- There was no reason to expect increased foaling rates based on current data.

Experience during the program’s initial 4 years suggested a database with individualized records of all horses (males, females, and juveniles) facilitated the observed outcomes. Improvements in data recording and treatment delivery over this period were ascribed to increased familiarity with both the environment and target population. Consequently, this method of immunocontraception was associated with providing an effective, humane, publicly acceptable, and practical alternative to the previous reliance on lethal, logistically demanding, or inhumane control methods.

## Figures and Tables

**Table 1:** Summary of pZP vaccine treatments of the Virginia Range mare population between April 2019 and December 2022.

	Mature Mares (≥1 years-old)			Immature Mares (<1 years-old)		
Year	Total (n)	Mares Vaccinated (n)	pZP Treatments (n)	Total (n)	Mares Vaccinated (n)	pZP Treatments (n)
2019	1528	760	1195	323	5	6
2020	1794	1070	1706	468	48	69
2021	1993	1202	1734	352	64	101
2022	1963	1257	1821	223	33	57

**Table 2:** Summary of the occurrence of darting-associated lesions characterized as either a granuloma or an abscess and their annual prevalence as a proportion of the total number of vaccines administered between 2019–2022.

	2019	2020	2021	2022
Granulomas	0	1	5	11
Abscesses	7	17	22	24
Granuloma prevalence	0.00%	0.06%	0.27%	0.59%
Abscess prevalence	0.58%	0.96%	1.20%	1.28%

**Table 3:** Total foal births summed by year and sex ratio (male/female/unknown gender). Additionally, total foal mortality and foals removed from the range are summarized by both year and gender.

Year	Total Foals	Dead	Removed
Gender	(M/F/N)	(M/F/N)	(M/F/N)
2019	392 (198/191/3)	67 (36/31/0)	6 (5/1/0)
2020	488 (238/247/3)	200 (98/102/0)	11 (8/3/0)
2021	267 (111/154/2)	157 (58/99/0)	4 (1/3/0)
2022	205 (96/108/1)	129 (57/72/0)	3 (3/0/0)

## Data Availability

The data that supported the findings of this study are available from the corresponding author upon reasonable request.
